# Perceptions of physical and occupational therapists on the utility of surface electromyography data in spinal cord injury rehabilitation

**DOI:** 10.1371/journal.pone.0351338

**Published:** 2026-06-15

**Authors:** Nadeen Al Awamry, Laura Seidelin, Alyssa Marino, Ethan Evans, Elizabeth Karam, Vishwa Kumar, Kristin E. Musselman, Anita Kaiser, José Zariffa

**Affiliations:** 1 Department of Physical Therapy, Temerty Faculty of Medicine, University of Toronto, Toronto, ON, Canada; 2 KITE Research Institute, Toronto Rehabilitation Institute - University Health Network, Toronto, ON, Canada; 3 Temerty Faculty of Medicine, Rehabilitation Sciences Institute, University of Toronto, Toronto, ON, Canada; 4 Institute of Biomedical Engineering, University of Toronto, Toronto, ON, Canada; 5 Edward S. Rogers Sr. Department of Electrical and Computer Engineering, University of Toronto, Toronto, ON, Canada; Polytechnic University of Marche: Universita Politecnica delle Marche, ITALY

## Abstract

**Purpose:**

Spinal cord injury (SCI) impacts physical, emotional, and social well-being, contributing to decreased quality of life and increased healthcare burden. Surface electromyography (sEMG), a non-invasive tool for measuring muscle activity, has demonstrated potential as a biomarker for recovery in SCI research, yet remains underutilized in clinical practice. Understanding how physical therapists (PTs) and occupational therapists (OTs) perceive the use of sEMG is necessary for integrating sEMG into post-SCI treatment and advancing personalized rehabilitation.

**Materials and methods:**

A cross-sectional, qualitative descriptive design was employed. Ten participants (9 PTs and 1 OT) were recruited through convenience sampling. Semi-structured interviews were conducted and analyzed inductively using a thematic analysis approach.

**Results:**

Two major themes were identified: 1) Perceived value of the use of electrophysiology and sEMG data in clinical practice. Participants valued sEMG as an adjunct assessment tool for providing objective feedback after incomplete SCI and setting goals during treatment. 2) Barriers and facilitators to implementing sEMG. Key barriers highlighted include the lack of training and standardized protocols. Continued training, resources, and educational support were key facilitators.

**Conclusion:**

PTs and OTs perceive sEMG as a valuable tool in SCI rehabilitation, but desire education and standardized protocols to support its clinical integration.

## Introduction

Spinal cord injury (SCI) is a significant and escalating health concern in Canada, resulting in lifelong consequences for those affected and substantial costs to the healthcare system. SCI can be traumatic (TSCI) in nature, occurring from external force, or non-traumatic (NTSCI), caused by factors such as tumours, vascular injury, or degenerative diseases [1]. In 2019, 1,199 cases of TSCI were reported in Canadian hospitals, a 37% increase from 2005, with over 30,000 individuals estimated to be living with TSCI [2].The average age of injury has also steadily increased, reflecting a shift in the SCI population [2]. These trends indicate that SCIs are a growing concern in Canada, as the number of injuries as well as rates of discharge into the community have continued to rise since 2005. Although incidence rates may appear relatively low in Canada, the economic and personal costs of SCI are extensive. Lifetime care costs exceed $2–3 million per individual, and the national economic impact of new TSCI cases is estimated at $2.67 billion annually [3,4].

Considering the complex effects of SCI, neurorehabilitation is essential for improving functional outcomes and quality of life (QoL). While the emphasis of post-SCI rehabilitation is centred on developing compensatory strategies that can maximize function in daily activities, there is a growing focus on neurological recovery [5]. To this end, an evolving model of care coined precision rehabilitation is gaining recognition. Precision rehabilitation applies principles of precision medicine to treatment and aims to tailor interventions to the individual by predicting which therapeutic approach will yield optimal outcomes based on patient-specific characteristics [6]. This approach relies heavily on measurable indicators called biomarkers to support data-driven, personalized decision making.

In neurorehabilitation, electrophysiology offers promising biomarker tools, due to its ability to directly assess the integrity of neural pathways for people with SCI [7]. Among these, surface electromyography (sEMG) is the most prevalent tool in SCI research [7]. sEMG is a non-invasive technique for recording muscle activity that can provide several types of information not available from commonly used manual examinations, such as the International Standards for Neurological Classification of Spinal Cord Injury (ISNCSCI). For example, sEMG has been used for prognostication of long-term recovery, providing biofeedback, understanding muscle activation and interlimb coordination, measuring dynamic motor control in gait, and tracking responses to therapy [8]. Additionally, it can detect muscle activation despite the absence of visible movement and is not affected by ceiling effects commonly found in clinical tests such as manual muscle testing [9–11]. sEMG can also assess small or difficult-to-measure muscle groups and provides high-resolution data on multiple muscles during functional tasks, making it a valuable tool in both assessment and treatment planning [11]. Given the extensive information that sEMG can provide, its accurate interpretation can have benefits for guiding rehabilitation care following SCI. Additionally, sEMG has the potential to be incorporated into therapy sessions because it requires simpler instrumentation and acquisition protocols than other modalities, such as motor evoked potentials [11].

Despite its potential benefits, sEMG is not commonly employed at the point of care. There is a gap in the literature regarding how physical therapists (PTs) and occupational therapists (OTs), healthcare professionals who work to improve patients’ functional abilities, perceive the use of sEMG in SCI rehabilitation. One study indicated technical requirements and lack of inclusion in PT/OT training programs as barriers to the increased use of sEMG in routine clinical practice [12]. Few studies have investigated the perspectives of health care professionals on the use of similar biomarker technologies in clinical practice [8,13]. The literature calls for more research to investigate the potential roles of sEMG in neurorehabilitation, as clinicians are reportedly eager to learn more about it and take steps toward implementing sEMG into their practice [8]. The perceptions of therapists regarding how sEMG may fit into their practice are crucial for successful translation. Their insights can inform future training, workflow design, and implementation strategies that align with the realities of clinical care.

This study explored the perceptions of PTs and OTs on the use of sEMG data in SCI rehabilitation. We aimed to 1) understand PTs’ and OTs’ perceptions about current and potential roles of electrophysiology in assessing patients with SCI or guiding treatments; and 2) understand if and how PTs and OTs might use sEMG data if it were available at the point of care.

## Methods

### Study design

This study adopted a cross-sectional, qualitative, descriptive methodology [14], using individual semi-structured interviews, to explore how Canadian OTs and PTs perceived the use of sEMG in SCI rehabilitation. A qualitative approach was particularly suitable for this inquiry, as it captured detailed participant insights, behaviours, and clinical reasoning that quantitative methods may not fully reveal. Specifically, qualitative descriptive studies provide rich, practical descriptions of participant experiences, which is critical in areas with limited existing research [15], such as PTs and OTs’ perceptions on the practicality and value of using sEMG data in SCI rehab contexts. Study procedures were approved by the Research Ethics Board of the University of Toronto (#47321), and all participants provided written informed consent. Participant recruitment took place between November 14, 2024 and June 16, 2025.

### Participant selection and sample size

To be eligible to participate in this study, all participants had to be either a licensed OT or PT working in Canada who had treated at least one SCI patient in the past year. To ensure data relevance and alignment with the research objectives, participants had to work in clinical settings where sEMG may be applicable, including inpatient, outpatient, or private practices. Participants had to be proficient in English to engage in the interviews. PTs and OTs who work solely in home care settings were excluded, as access to sEMG in these environments is limited. This inclusion-exclusion approach ensured that participants had sufficient experience and context to provide meaningful input on the practical use of sEMG in SCI care.

A convenience sampling approach [16] was used during the recruitment process. Recruitment materials—including posters and emails — were distributed nationally via the research team’s professional networks and institutions (e.g., clinician colleagues), the Canadian ABT Community of Practice, the Neurosciences Division of the Canadian Physiotherapy Association, and the Canadian Association of Occupational Therapists. The proposed sample size was 10–12 participants, based on the concept of information power, which suggests that the more relevant and richer the information of the sample is, the smaller the sample size needed. The size of a sample with sufficient information power [17] is determined by the aim of the study, the sample specificity, the use of an established theory, the quality of the dialogue, and the analysis strategy. For this study, the aim is narrow, and the sample is moderately specific given the inclusion and exclusion criteria. The Unified Theory of Acceptance and Use of Technology (UTAUT) framework was used to develop the interview guide. Although the interviewers had minimal prior experience with qualitative research, pilot interviewing was done before data collection to help strengthen the qualitative interviewing technique before any semi-structured interviews. This study used a cross-case analysis strategy. In summary, three of the five factors influencing information power suggested a small sample was required, defending the use of a moderate sample size of 10–12 participants.

### Data collection

Data collection was informed by the UTAUT framework. This model helped to guide the exploration of how PTs/OTs accept and use sEMG, focusing on constructs such as performance expectancy, effort expectancy, social influence, and facilitating conditions [18]. The interview guide was developed using the UTAUT framework and included open-ended questions exploring:

- Clinical experiences in SCI rehabilitation
- Assessment practices related to neurological recovery
- Familiarity with electrophysiology
- Perspectives on the potential clinical role of sEMG in assessment and treatment

Before the interview, participants completed a demographic questionnaire that collected general characteristics, including each participant’s age, gender, current employment status, current job title, work setting, province of residence, and number of years working in SCI rehabilitation. Data was then gathered through semi-structured interviews conducted individually via Zoom. Semi-structured interviewing methods allowed flexibility and reciprocity between the interviewer and the participants. This enabled a discussion in which the interviewer could ask improvised probing and follow-up questions based on participant responses and could create space for participants’ individual expression [19]. Each interview lasted approximately 45–60 minutes and was conducted by the same member of the research team to provide consistency between interviews. Reflexive notes were documented by another team member who was present for each interview [20]. Interviews were audio-recorded and transcribed verbatim.

### Data analysis

Interview data was analyzed using codebook thematic analysis, an iterative and inductive method that allowed researchers to identify patterns and themes from the data. The analysis followed the six-step DEPICT model [21], which promotes collaborative and transparent coding practices:

1. **Dynamic Reading**: Each transcript was reviewed by at least two team members, who noted initial impressions and emerging patterns.
2. **Engaged Codebook Development**: The team discussed findings to develop a preliminary inductive codebook, piloted on all transcripts and refined through consensus.
3. **Participatory Coding**: All researchers helped to code each of the transcripts using the final codebook, with ongoing team meetings to ensure alignment. All changes were tracked in an audit trail [22].
4. **Inclusive Reviewing and Summarizing**: The team reviewed coded data, summarized the codes, and identified significant quotes and silences.
5. **Collaborative Analyzing**: Team discussions focused on identifying and labelling themes. Discussions continued until a consensus was reached.
6. **Translating**: The final themes were interpreted and aligned with the study objectives for reporting in the results section.

NVivo 15 software was used to facilitate data management and analysis.

### Trustworthiness

Trustworthiness—defined by Shenton [23] as a measure of the confidence one can have in the accuracy and truthfulness of the research—was achieved through several strategies. Shenton described trustworthiness as a combination of credibility, transferability, dependability, and confirmability, which contribute to the overall quality of qualitative research [23]. To begin, researchers included a diverse sample of OTs and PTs across various settings (e.g., hospitals, clinics) that helped to capture a broader range of perspectives, contributing to the depth and transferability of the findings [24]. From here, the data collection and analysis were carried out by individuals with varying levels of experience in SCI rehabilitation and qualitative research. This diversity helped reduce potential bias during the interpretation of results [24]. Third, multiple authors (NA, EE, EK, VK, AM, LS) actively participated in the data analysis process, receiving ongoing guidance and feedback from senior researchers (AK, KEM, JZ) to ensure alignment with the study framework and objectives. Next, the use of verbatim participant transcripts, reflexive notes, and previous research on EMG data in SCI rehabilitation further enhanced the credibility and transferability of the findings [23]. Finally, verbatim quotes were included to illustrate and support the identified themes.

## Results

### Participant characteristics

The study included 10 participants, the majority of whom were women and PTs Table 1. Participants represented three provinces in Canada and had a broad range of experience working with the SCI population. Participants worked across a variety of settings, including acute care, rehabilitation hospitals, private practice, and not-for-profit community clinics in urban and suburban settings. Nearly all participants (n = 8) described most frequently working with both complete and incomplete SCI patients.

**Table 1:** Participant Demographics.

Variable	Participant Characteristics (n = 10)
**Age** (median)	33.5
**Current job title**	
Physical Therapist (PT)	9
Occupational Therapist (OT)	1
**Gender**	
Woman	8
Man	2
**Province of residence**	
British Columbia	2
New Brunswick	1
Ontario	7
**Employment status**	
Full-time	9
Part-time	1
**Years of Practice**	
0-3 years	2
4-6 years	3
6-8 years	1
8-10 years	1
10 + years	3
**Years of experience working with patients with spinal cord injury**	
0-3 years	3
4-6 years	2
6-8 years	3
8-10 years	0
10 + years	2

### Themes

Two overarching themes were identified: 1) Perceived value of the use of electrophysiology and sEMG data in clinical practice, and 2) Barriers and facilitators to implementing sEMG. Both themes were further divided into sub-themes to capture the nuanced perspectives and experiences of the PTs and OT, allowing for a more thorough understanding of how they conceptualize the role of sEMG data in practice and what factors influence its adoption. The sub-themes reflect distinct yet interconnected dimensions of each main theme, such as PTs/OT’s reliance on conventional assessments versus their openness to new technologies, or the specific challenges and enabling conditions that shape implementation. This thematic structure enables a comprehensive examination of both the potential benefits and practical considerations surrounding the use of sEMG data in rehabilitation settings Table 2.

**Table 2:** Themes and sub-themes.

Theme 1: Perceived value of the use of electrophysiology and sEMG data in clinical practice	Sub-theme 1.1: PTs and OTs prioritize manual and functional assessments to guide their treatment plans
Sub-theme 1.2: PTs and OTs have experience with electrical stimulation for treating muscle function, with limited exposure to electrophysiology as an assessment tool
Sub-theme 1.3: Recognition for the supplementary value of sEMG if it provides additional insight
Theme 2: Barriers and facilitators to implementing sEMG	Sub-theme 2.1: Barriers limiting the use of sEMG in clinical practice
	Subtheme 2.2: Facilitators for effective implementation of sEMG into clinical practice

OTs: occupational therapists; PTs: physical therapists; sEMG: surface electromyography.

#### Theme 1: Perceived value of the use of electrophysiology and sEMG data in clinical practice.

This theme examines the perspectives of PTs and OTs on the utility of electrophysiological tools, particularly sEMG, in rehabilitation. While the PTs and OT prioritized conventional functional assessments, they acknowledged the supplementary role of electrophysiology for specific cases, such as neuromuscular retraining. The theme is divided into three sub-themes.

Sub-theme 1.1: PTs and OTs prioritize manual and functional assessments to guide their treatment plans.

Participants described relying heavily on traditional physical and occupational therapy assessments, manual muscle testing (MMT), proprioception exams, and functional outcome measures to guide treatment. One OT described how they assess neurological recovery:

*I would say a lot of it’s client reported...So like, ‘Oh, I noticed I can touch my nose this week versus like last week I couldn’t get my arm up to here and like things like that, right?’ And then...it might probe me to inquire a little bit further...but certainly a lot of it would be observed too. (OT1)*

One community PT highlighted the importance of individualized assessments to accurately detect change in patient status:

*The reassessing point is big. Because sometimes it feels like there’s no change. But it’s because you...haven’t been creative enough with how you’re assessing them...What you learn in school, what you learn early in your practice...it’s not enough. You’ve got to think of what’s another way you can assess them...you’re going to learn your own ways with each person on how to best assess and treat them.(PT6)*

Additionally, PTs/OT incorporate information from the interdisciplinary team and medical charts to contextualize their assessment findings. Participants in outpatient settings noted limited access to patients’ information from previous or other clinical settings, which increased their reliance on clinical observations to monitor progress and guide treatment.

Treatments often consist of task-specific mobility exercises, such as bed and wheelchair transfers, further illustrating why conventional assessments, which break down these actions into measurable components like strength, balance, and coordination, are central to treatment decisions. When probed on what information they prioritize in their treatment and assessment of patients, one PT noted, *“When you work in inpatient rehab, it’s really about the functionality of everything” (PT1).* Their focus remains on improving independence, with conventional interventions tailored to each patient’s functional needs.

Sub-theme 1.2: PTs and OTs have experience with electrical stimulation for treating muscle function, with limited exposure to electrophysiology as an assessment tool.

Many participants employ electrophysiology therapeutically via the delivery of electrical stimulation (e.g., functional electrical stimulation (FES), neuromuscular electrical stimulation (NMES), transcutaneous electrical neurostimulation (TENS)), especially for SCI patients, to preserve muscle mass, improve range of motion, and support bone density. One therapist described how patients *“use the FES bike to maintain muscle mass and function as they get older”* (PT8). Participants recognize the therapeutic value of using electrophysiology and its favourable outcomes for treating patients. However, few participants use electrophysiology systematically for evaluation and view it primarily as a therapeutic tool. Some participants, like this PT, repurpose electrical stimulation for informal assessments and to monitor patient progress:

*So sometimes, especially in cauda equina...type presentations, I’ll use stim, even just as an assessment tool, to see if there is a connection; if it is stimmable…and then, we can redo that later because often one week it isn’t and then it suddenly is.* (PT4)

This adaptive approach reflects a shift in how some PTs and OTs view electrophysiology, not only as a therapeutic tool, but also to potentially support and enhance clinical assessment.

Sub-theme 1.3 Recognition for the supplementary value of sEMG if it provides additional insight

Despite a lack of hands-on experience with sEMG, participants demonstrated a recognition of its current and potential roles and how it could supplement their treatment. This includes perceived utility of sEMG for patient education, biofeedback, gaining insight into patient status, and guiding treatment and assessment. Some participants highlighted the perceived use of sEMG as an investigative tool to identify muscular activation, or lack thereof; an area where conventional assessments fall short and how application of sEMG data may potentially inform the use of electrical stimulation:

*I think it would be interesting to see like...in someone who has weakness in a particular muscle, if there is any sort of connection to like the neuromuscular connection there, because sometimes...it’s hard to tell if…there’s nothing there or if it’s just like really weak. So...I feel like that would be good to…quantify it objectively. (PT9)*

Some participants also described the value of sEMG in informing the allocation of interventions:

*I think for education or...evaluating whether or not certain types of electromuscular stimulation will be effective or not, or...if somebody’s got a bit of a lower motor neuron injury presentation, helping to allocate what your interventions are a bit more effectively. (PT5)*

However, the perceived utility of sEMG data varies depending on the patient population. Participants described specific patient cases where sEMG would be particularly useful. Some PTs/OT perceived that patients with incomplete SCI may benefit from sEMG as it may help identify potential neurological recovery. Participants perceived minimal benefit, even potential discouragement, for individuals with complete SCI or little to no muscle innervation. They also described specific time points in rehabilitation where sEMG application could be most beneficial. Some participants perceived that this may not be as useful for patients with chronic SCI. Some participants, like this PT, noted that early in rehab, sEMG can provide critical baseline information:

*...earlier in their stages of rehab would be a little bit more beneficial just to see...what muscles are able to get contraction and...innervation...but long-term effects, I feel like it would be good to see if the treatment is...kind of maintaining their strength, their activation and seeing like all the muscle activity as they progress through treatment. (PT8)*

The timing of sEMG use becomes even more crucial when working with patients after nerve transfer surgeries; one participant identified an ‘optimal window’ during which neural connections begin to form—often around six to nine months post-surgery—when sEMG can serve as a motivating and guiding tool through incorporating biofeedback: *“That is supposed to be the more like optimal time where then they’re able to actually try to work on actively contracting the muscle and get that feedback.”* (PT5)

The potential for sEMG to motivate patients by revealing subtle signs of recovery was also noted, particularly for acute care settings. As one PT described, sEMG can serve as an educational and motivational tool for patients with incomplete injuries where progress is less predictable:

*I think it… just becomes another tool in your toolbox that you can use to provide education for your patients,…set realistic goals...and expectations of their functional outcome, especially the incomplete injuries, which can be a bit of a coin toss. I think it could be a really good motivator for patients because a lot of people, after spinal cord injury, are very upset, down...They’re dealing with a big change to their life. And so being able to give them some tactical physical data that something is going on might be helpful to their...mental health and to their motivation to participate in rehab.* (PT3)

#### Theme 2: Barriers and Facilitators to Implementing sEMG.

This theme examines both the challenges and enablers influencing the adoption of sEMG in rehabilitation settings. Participants identified key barriers, including skepticism about clinical utility, lack of standardized protocols, and insufficient training, while also highlighting facilitators such as a strong desire for education, evidence-based guidelines, and internal advocacy. The theme is divided into two sub-themes.

Sub-theme 2.1: Barriers limiting the use of sEMG in clinical practice

Participants identified internal and external constraints that limited the adoption of sEMG into their practice. Internally, some struggled with skepticism about its clinical utility, questioning whether it meaningfully impacted their treatment planning. One PT noted, “... *But again, it rarely adds anything that I don’t know clinically. I usually can see if there’s a C5 palsy; I don’t need the EMG study to show me the C5 [laughs] isn’t working.” (PT4)*

Without clear evidence of its practical value, PTs/OT would not rely on sEMG findings to shape their care decisions. Many pointed to broader issues: a lack of knowledge, formal training, and uncertainty about how to interpret the data in a way that meaningfully guides treatment. As one PT explained, they had “*never learned anything in school or like through my time about…using EMG data to then help guide my treatment plan...”* (PT1)*,* Many described the general lack of exposure to sEMG in their academic and clinical training leading to a lack of confidence and uncertainty on how to apply it in practice. The absence of standardized guidelines left participants hesitant about how to proceed.

External barriers compounded these challenges. In fast-paced rehabilitation settings, time and resources for learning and implementing new technology are limited. *“...Some limitations and drawbacks could be...the setup time and...complexity of it...If they don’t see...instant benefit from being set up on it, a lot of people will find that, if there’s an extended setup time, it takes away from their session.” (PT7)* Participants also described environments where adding new tools like sEMG often felt unsupported, leaving little space to build comfort and competence with their use.

Subtheme 2.2: Facilitators for effective implementation of sEMG into clinical practice

The PTs and OT highlighted the need for targeted education sessions, accessible resources, and ongoing training to facilitate sEMG use. One PT emphasized the impact that direct instruction would have on their confidence and competence:

*I think definitely would be beneficial to have like an education session if there was…a specific vendor or someone who makes that kind of device,…having them come into the clinic to just educate the therapist on how to use it and like what you’re looking for, that would be beneficial.* (PT9)

Participants also envisioned clear, accessible resources, including fact sheets, online databases, or courses to support clinical decision-making and bridge knowledge gaps. Importantly, they emphasized that adoption of sEMG depends on evidence of its clinical validity, with one PT stating: “*If it is the way that...the field is moving in terms of physiotherapy and scope of practice, like, is this something that can really provide valuable information and is that proven in the literature?”* (PT2)

Finally, participants described the power of having a clinical champion; someone who could advocate for the technology, support colleagues, and drive adoption across the team. One PT reflected on this:

*Sometimes, if someone has been using it and then they are the ones that champion the use in the center, like that can really have a huge impact to have just one person that really stands behind it and then encourages others to use it or is able to help with questions or train people.* (PT2)

## Discussion

This study explored the perceptions of nine PTs and one OT regarding the utility of sEMG data in SCI rehabilitation. Drawing on their clinical experience, participants expressed their thoughts on the use of this technology in future practice. The findings reveal that participants predominantly rely on conventional functional assessments (Sub-theme 1.1) as well as therapeutic electrophysiology but have limited systematic experience with sEMG as an evaluative tool (Sub-theme 1.2). Despite this, PTs/OT recognize sEMG’s potential to supplement practice, particularly for neuromuscular insight, patient motivation, and guiding interventions in specific populations (Sub-theme 1.3). However, adoption is hindered by barriers such as skepticism, lack of training, and unclear protocols (Sub-theme 2.1). Participants also identified that facilitators such as education, evidence-based guidelines, and clinical champions could bridge this gap (Sub-theme 2.2). The results of this study highlight a tension between sEMG’s perceived value and practical implementation challenges, setting the stage for a discussion on how to align its utility with clinical workflows and evidence-based rehabilitation paradigms.

Participants agree that sEMG data has the potential to serve as a useful tool through its ability to provide objective data about motor innervation, specifically when ‘traditional’ assessments may lack sensitivity or fail to capture more subtle changes in motor activity. This supports other findings in the literature that confirm participants’ perception that sEMG data can provide quantitative evidence to support treatment decisions [8]. Feldner and colleagues reported that participants in their study identified the potential role of sEMG data in prognosticating recovery and with that its ability to provide secondary quantitative information that could help guide clinical decisions [8]. Our findings extend this narrative by highlighting the specific value our participants placed on sEMG data as a complementary tool rather than a replacement. There was perceived value of sEMG not only being used as a diagnostic tool for neurological recovery, but also to support patient engagement, particularly through real-time biofeedback. The ability of sEMG to make otherwise invisible neural activity visible to both the patient and the therapist was perceived as a tool that could improve understanding of motor control and assist in setting realistic treatment goals. This sentiment aligns with other work in which PTs and OTs working with adults and children with neurologic conditions noted that in their clinical exposure to sEMG technology, patients reported enjoying biofeedback as an encouraging tool to show signs of progress [8]. In addition to identifying the potential valuable roles of sEMG in practice, participants also described their familiarity with other forms of electrophysiology, such as FES and NMES. This foundational knowledge of the use of related technologies in clinical practice may also help to facilitate the implementation of additional neurotechnologies such as sEMG. However, it should be noted that this shift in mindset would be dependent on successful behaviour change on an individual clinician level [25].

Despite the recognition of the value of sEMG data, some participants also expressed skepticism regarding its clinical utility. This skepticism is echoed by research showing that sEMG does not always capture meaningful clinical change. For example, Korczyński et.al [26] found that clinical improvements captured by clinical outcome measures such as the Walking Index for Spinal Cord Injury (WISCI-II) were not reflected in average peak muscle amplitude (AMA) sEMG scores. Our participants also conveyed clear, consistent concern regarding the barriers that would affect sEMG implementation. Most significantly, the PTs and OT expressed the need for standardized clinical protocols and hands-on training. Many of these barriers have been previously identified in the literature. For example, a study on barriers and future considerations for the use of sEMG in SCI rehabilitation found that the major obstacle to implementing sEMG data in practice was clinicians’ lack of confidence, lack of exposure, and limited foundational knowledge within both their educational and professional curricula [27]. It is evident that across the spectrum, clinicians feel as though these barriers are not solely technical in nature, but they are also systematic and educational. Similar key barriers have been described in alternative studies evaluating the utility of other neurotechnologies, such as FES and NMES, in practice [28]. These findings highlight the idea that entry to practice education for both PTs and OTs has a strong focus on manual skills, with clinicians getting limited exposure to technology and its use in practice [28]. Additionally, all participants identified that the potential future use of sEMG data in SCI rehabilitation practice would require coordinated efforts within workplace infrastructure and would be most effective with the help of interdisciplinary collaborations. This concern is similar to one that was presented by Pilkar and colleagues, which discussed the use of sEMG in SCI and the barriers to implementation in clinical practice [27]. The OT and PTs in this study highlighted that the processing and interpretation of sEMG data would require knowledge that goes beyond their current skill set and that successful analysis of sEMG data would require seeking out other professionals with this expertise [8]. Similarly, Cappellini et al [29], who surveyed Italian neurorehabilitation professionals found that while the majority of participants identified educational and training deficits as the primary barrier, others identified the time-consuming nature of sEMG and the lack of a multidisciplinary team, amongst other barriers, as practical challenges to integration of sEMG into clinical workflow. Altogether, these studies reinforce the concerns raised by our participants on the practical barriers associated with integrating sEMG into routine practice.

The findings of this study suggest a need for stronger evidence supporting the clinical utility of sEMG.. Clinical utility is often evaluated by considering the cost, portability, training required, and time required to complete the assessment [30]. Current participants displayed skepticism regarding the relevance of implementing new technology into their approach to practice, preferring their current methods. However, participants consistently reported desiring to learn more about sEMG and how to use sEMG data, which is consistent with existing research [9]. Considering these factors, there appears to be a need for more clinic-friendly sEMG devices and stronger evidence supporting sEMG’s clinical utility. In this study, there were several potential benefits identified for integrating objective tools such as sEMG into routine clinical practice to complement clinical decision making, support patients on an individualized basis, and assist them in engaging more meaningfully in their rehabilitation journeys. The insights of this study are particularly relevant as the healthcare system moves towards a precision medicine model, one that tailors assessment and treatment interventions based on personalized variables [6].

Following an established increase in the clinical utility of sEMG, there is a significant need for further research involving the creation of educational resources and practical experiences of PT/OTs regarding sEMG data. Several participants emphasized a desire for evidence-based guidelines on how to use sEMG, training courses for using sEMG and interpreting data, and standard protocols for how to use sEMG data in treatment planning for SCI populations. Developing and testing these potential educational resources is the next step towards effective implementation of sEMG data in clinical practice [31]. Additionally, future studies could provide PT/OT participants with sEMG data on their patients and later interview them to discuss how it was used and its impact, if any, on their patients’ rehabilitation. Findings from this study outlined the ways PTs/OTs could imagine using sEMG data, and an important next step would be observing these methods of use in practice.

These results elaborate on the existing evidence and understanding of barriers and facilitators to the use of sEMG in clinical practice, which is a key step in knowledge translation. Identifying barriers has been identified as a necessary phase when moving towards knowledge application across many frameworks [31]. By highlighting the perceptions of the PTs/OT in this study, this research can contribute to a deeper understanding of both the enablers and barriers to implementation. A deep understanding comes from qualitative inquiry, which we have conducted in this study [32].

This study has some limitations to acknowledge. First, only one OT was interviewed, which limits the generalizability of these findings. As we primarily recruited interested study participants through the research teams’ professional networks, and the research team consisted predominantly of physical therapy students, our recruitment of OTs was limited. Further, the geographic location of where participants were located was mostly in Ontario, which may create limitations in generalizability across Canada and different rehabilitation systems. In addition to these limitations, it should also be noted that there are sources of potential bias inherent to qualitative research that may have impacted this study’s results. One of the main sources of potential bias is social desirability. It is possible that participants responded to interview questions in a way that they deemed socially acceptable [33]. This could present as a limitation as participants’ responses may have been skewed from their actual reality [33].To mitigate this bias, the interviewer strived to maintain a neutral tone throughout each of the interviews to limit the perception that a certain response was well regarded by the interviewer. The observer-expectancy effect could also be considered a potential limitation to this qualitative study. This effect explains that participants may respond in a way that they perceive as ‘correct’ in hopes of having their response be well-regarded by the interviewer [34]. The participants in this study may have responded or behaved in a way that aligned with the researchers’ views or desired outcomes. To limit this effect, the interviewers relied on the interview guide, which framed questions in a non-leading way and ensured questions were delivered in a neutral way to limit the researcher’s views from influencing the responses.

## Conclusion

PTs and OTs working with individuals with SCI perceive sEMG to be a potentially useful adjunct to their current treatment and assessment approaches. While they currently have limited experience using electrophysiological tools for assessment purposes, including sEMG, they are eager to learn more. PTs and OTs desire educational resources and evidence-based protocols regarding how to use sEMG data to enhance their patients’ care. The findings from this study emphasize the need for future research and development into the clinical utility of sEMG data and the development of educational resources for PTs and OTs to improve perceived competence and confidence. These insights are especially pertinent as the healthcare system advances toward a precision medicine model, one that aims to customize assessment and treatment strategies based on individual-specific measurable indicators [6].
